# Cisplatin fails to induce puma mediated apoptosis in mucosal melanomas

**DOI:** 10.18632/oncotarget.3195

**Published:** 2015-03-26

**Authors:** Marie Kristin Fritsche, Veronika Metzler, Karen Becker, Christian Plettenberg, Clemens Heiser, Benedikt Hofauer, Andreas Knopf

**Affiliations:** ^1^ Technische Universität München, Hals-Nasen-Ohrenklinik und Poliklinik, 81675 München, Germany; ^2^ Universität München, Institut für Allgemeine Pathologie und Pathologische Anatomie, 81675 München, Germany; ^3^ Heinrich Heine Universität Düsseldorf, Hals-Nasen-Ohrenklinik, 40225 Düsseldorf, Germany

**Keywords:** melanoma, sinonasal, survival, p53, cell-cycle

## Abstract

**Objectives:**

Mucosal melanomas (MM) are aggressive subtypes of common melanomas. It remains unclear whether limitations in their resectability or their distinctive molecular mechanisms are responsible for the aggressive phenotype.

**Methods:**

In total, 112 patients with cutaneous melanomas (CM) and 27 patients with MM were included. Clinical parameters were analysed using Chi square, Fisher exact and student's *t*-test. Survival rates were calculated by Kaplan–Meier. Analysis of p53, p21, Mdm2, Hipk2, Gadd45, Puma, Bax, Casp9 and Cdk1 via quantitative PCR and immunohistochemistry (IHC) was performed. *TP53* induction after cisplatin treatment was analysed in 10 cell lines (melanocytes, four MM and five CM) using western blot (WB) and qPCR.

**Results:**

The overall/recurrence-free survival differed significantly between MM (40 months and 30 months) and CM (90 months and 107 months; *p* < 0.001). IHC and WB confirmed high p53 expression in all melanomas. Hipk2 and Gadd45 showed significantly higher expressions in CM (*p* < 0.005; *p* = 0.004). QPCR and WB of wild-type cell lines demonstrated no differences for p53, p21, Mdm2, Bax and Casp9. WB failed to detect Puma in MM, while Cdk1 regulation occurred exclusively in MM.

**Conclusions:**

The aggressive phenotype of MM did not appear to be due to differential expressions of p53, p21, Mdm2, Bax or Casp9. A non-functional apoptosis in MM may have further clinical implications.

## INTRODUCTION

In Europe, 18 of 100,000 people are annually diagnosed with malignant melanoma, and the incidence is continuously increasing. Earlier detection by intensive clinical efforts have led to an enhanced five-year survival rate of 89%–94% [1]. Mucosal melanomas represent an infrequent subtype representing 1% of the overall cohort [2]. The majority of MM originates in the sinonasal region. Other tumour sites such as the oral cavity, uvea, or the urogenital and gastrointestinal tracts occur infrequently [3]. In contrast to their cutaneous counterparts, MM present a highly aggressive subtype with a poor five-year survival rate of approximately 17% [4]. The sinonasal region is a difficult site to access surgically, often preventing a radical surgical approach with sufficient R-status. Therefore, locoregional recurrent disease might be a possible explanation for the limited survival. Molecular mechanisms underlying the highly aggressive phenotype remain unclear. While the aetiology of MM is still unclear, molecular changes that may contribute to tumour development in CM are widely discussed [5–10]. While *TP53* is mutated in many solid tumours, mutations in CM and MM are rare [11]. However, accumulation of wild-type p53 can be detected in the majority of CM and MM. The mechanisms explaining the p53 protein stabilisation remain unclear. Mutations in p53-modifying proteins Mdm2 (*mouse double minute 2*) and Hipk2 (*homeodomain-interacting protein kinase 2*) can be excluded [12]. Cellular stress, such as damage induced by UV light and cytotoxic agents, leads to p53 stabilisation by blocking its degradation through Mdm2 and posttranslational modifications [13]. Once p53 is activated, it functions as a transcription factor in the expression of a broad variety of target genes involved in cell cycle regulation, DNA repair and apoptosis [14]. *MDM2* is a direct target gene, which mediates p53 degradation. Under stress conditions, this feedback loop is inhibited by phosphorylation of p53 on Thr18 through Ck1 (*casein kinase 1*) thereby disrupting Mdm2-p53 binding [15]. Stabilised and activated p53 can induce cell cycle arrest by enhancing the expression of p21 which itself inhibits Cdk1 (*cyclin dependent kinase 1*) and other Cdks, decelerating cell cycle progression [16]. The influence of p53 in DNA repair was explored by inducing the expression of *GADD45A* (*growth arrest and DNA damage-inducible 45*). In addition, *GADD45A* is involved in cell cycle regulation, survival and apoptosis [17]. The p53-induced apoptosis is accomplished by expressing the target genes *PUMA* (*p53 upregulated modulator of apoptosis*) and *BAX*. Through its BH3 domain, Puma binds the anti-apoptotic proteins Bxl-X_L_ and Bcl-2 prohibiting their inhibitory function on Bax [18]. Active Bax permeabilizes the mitochondrial membrane, releasing cytochrome c. This leads to caspase activation resulting in apoptosis [19]. Despite missing *TP53* mutations in CM and MM, the *in vivo* and *in vitro* response of classical p53-inducing chemotherapeutic agents such as cisplatin is poor [20].

We give a detailed assessment of the clinical characteristics of 139 patients with malignant melanoma of the head and neck, including 112 CM and 27 MM. To investigate whether or not the aggressive phenotype in MM is due to an aberrant p53 pathway we analysed the protein and mRNA expression of p53 and its targets. Formalin-fixed, paraffin-embedded (FFPE) samples of 40 patients with head and neck CM and MM were differentially analysed using immunohistochemistry and real-time (RT-) PCR (qPCR). Furthermore, we investigated the functional integrity of p53 in CM and MM melanoma cell cultures by cisplatin incubation. Experimental data was put into clinical context.

## RESULTS

### Epidemiology

A total of 139 patients with malignant melanoma of the head and neck, treated in the Department of Otorhinolaryngology, Technical University Munich, were included in the current study. There were 112 patients with CM and 27 patients with MM. Twenty-five patients with MM demonstrated a sinonasal tumour manifestation, and two patients had tumours in the oral cavity. Patients with CM were significantly younger (mean age of 58 years, SD: 15) than patients with MM (70 years, SD: 12, *p* < 0.001). Concordant with the UICC classification system, the majority of MM was classified as T3 tumours. At the time of diagnosis two patients (7%) with MM showed locoregional lymph node involvement, as compared to 15 patients (13%) with CM (*p* < 0.396). Lymph node involvement in MM exclusively occurred in oral MM. In contrast, MM tended to exhibit less frequent distant metastases at the time of diagnosis (*p* < 0.064) (Table 1).

**Table 1:** Epidemiological data of mucosal melanoma and cutaneous melanoma MM Mucosal melanoma; CM Cutaneous melanoma

*n*	All 139	MuM 27	CM 112	*p*-value
Age at diagnosis (SD)	60 (15)	70 (12)	58 (15)	< 0.001
Sex (f/m)	65/74	16/11	49/63	= 0.126
T1	24	0	24	< 0.032
T2	22	0	22	< 0.012
T3	25	21	15	< 0.001
T4	20	6	14	< 0.198
Tis	19	0	19	< 0.022
Tx	29	0	18	< 0.005
N+	17	2	15	< 0.396
M+	13	0	13	< 0.064

### Survival analysis

**Figure 1:**
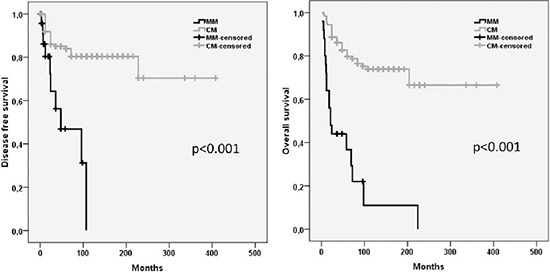
Kaplan–Meier estimates of the disease free and overall survival in patients with MM and CM MM Mucosal melanoma; CM Cutaneous melanoma

Recurrent disease was demonstrated in 10 (40%) MM and 30 (19%) CM (*p* < 0.001). The mean disease-free survival time in MM was significantly reduced (30 months) when compared with CM (107 months) (*p* < 0.001). After a mean follow-up of 89 months, the median overall survival (OS) differed significantly between both groups (MM: 40 months; CM: 99 months; *p* < 0.001) (Figure 1).

### p53 mutation status in MM cells and CM cell lines

Sequence analysis of the DNA-binding domain, comprising exon 5–8, of *TP53* revealed no aberration in MM cells (TU-MM1-TU-MM4). The identification of aberrations in CM cell lines (one missense mutation SK-MEL 3: R267W and one frameshift deletion IGR-37: C229DelTG) was previously described [21]. Whereas IGR-37 expresses mutant transcript only, SK-MEL 3 retains *p53* wild-type transcript. Sequence analysis revealed wild-type p53 in SK-MEL 30, MEL JUSO and COLO-849 (Data not shown).

### Proteins involved in the p53 pathway

Immunohistochemical staining revealed p53 positivity in 94% of the CM and 80% of MM with a significantly higher expression in CM. Mdm2 that is directly involved in the p53 protein stabilisation was positive in 71% of MM and 81% of CM without statistical significance. Immunohistochemical staining revealed a striking difference of Hipk2 staining patterns between the groups (*p* < 0.005). Hipk2, responsible for p53^Ser46^ phosphorylation therefore mediating enhancement of promoter-specific DNA binding, was negative in 88% of MM, whereas 44% of CM showed Hipk2 positivity. Despite the high number of p53-positive cases, the majority of MM (58%) and CM (63%) showed a weak p21 staining. CM demonstrated a significantly increased Gadd45a staining in 100% of analysed samples. Ninety-two percent of MM stained positive for Gadd45a (*p* = 0.004). Of the CM cases, 94% stained positive for Puma and 62% were positive for Bax; however, only 78% of the MM were positive for Puma and 42% for Bax. The observed tendency failed to reach statistical significance (*p* = 0.08; *p* = 0.09). In contrast to the high expression of pro-apoptotic proteins, the vast majority of CM (92%) and MM (81%) were negative for Casp9. Cell-cycle regulating protein Cdk1 was detected in 38% of CM and 63% of MM (Table 2).

**Table 2:** Immunhistochemical data of proteins involved in the p53 pathway MM Mucosal melanoma; CM Cutaneous melanoma

		<10%	11–30%	31–70%	>70%	*p*-value
		*n* (%)	*n* (%)	*n* (%)	*n* (%)	
**p53**	**MM (*n* = 24)**	5 (21)	5 (21)	4 (17)	10 (42)	< 0.04
	**CM (*n* = 16)**	1 (6)	2 (13)	0 (0)	13 (81)	
**p21**	**MM (*n* = 24)**	14 (58)	6 (25)	4 (17)	0 (0)	= 0.58
	**CM (*n* = 16)**	10 (63)	2 (13)	2 (13)	2 (13)	
**Mdm2**	**MM (*n* = 24)**	7 (29)	1 (4)	5 (21)	11 (46)	= 0.68
	**CM (*n* = 16)**	3 (19)	0 (0)	7 (44)	6 (38)	
**Hipk2**	**MM (*n* = 24)**	21 (88)	2 (8)	1 (4)	0 (0)	< 0.005
	**CM (*n* = 16)**	9 (56)	1 (6)	2 (13)	4 (25)	
**Gadd45**	**MM (*n* = 24)**	8 (33)	2 (8)	3 (13)	10 (42)	= 0.004
	**CM (*n* = 16)**	0 (0)	1 (6)	1 (6)	14 (88)	
**Puma**	**MM (*n* = 24)**	5 (21)	2 (8)	1 (4)	16 (67)	= 0.08
	**CM (*n* = 16)**	1 (6)	0 (0)	0 (0)	15 (94)	
**Bax**	**MM (*n* = 24)**	14 (58)	1 (4)	4 (17)	5 (21)	= 0.09
	**CM (*n* = 16)**	6 (38)	0 (0)	2 (13)	8 (50)	
**Casp9**	**MM (*n* = 24)**	22 (92)	2 (8)	0 (0)	0 (0)	= 0.14
	**CM (*n* = 16)**	13 (81)	1 (6)	1 (6)	1 (6)	
**Cdk1**	**MM (*n* = 24)**	15 (63)	4 (17)	3 (13)	2 (8)	= 0.38
	**CM (*n* = 16)**	6 (38)	5 (31)	5 (31)	0 (0)	

### Quantitative PCR of the p53 pathway in FFPE tumour samples

**Figure 2:**
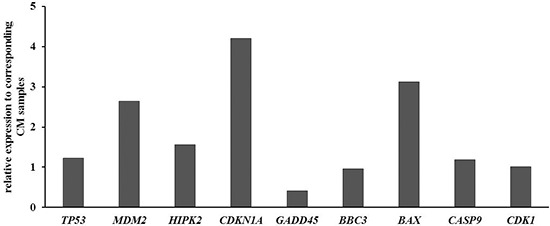
Quantitative PCR of the p53 pathway in FFPE tumor samples Results were normalized to GAPDH and shown as fold induction compared to CM. MM: Mucosal melanoma; CM Cutaneous melanoma.

QPCR of FFPE tumour samples did not show significant differences in the expression of *TP53*, *BBC3*, *CASP9*, *BCL2A1*, and *CDK1*. *HIPK2*, *MDM2*, *CDKN1A*, and *BAX* mRNA expression was increased in MuM demonstrating differences from 2.6- to 4.2-fold. *GADD45* showed 2.4-fold decreased expression in MM (Figure 2).

### Quantitative PCR of the p53 pathway in MM cells and CM cell lines

**Figure 3:**
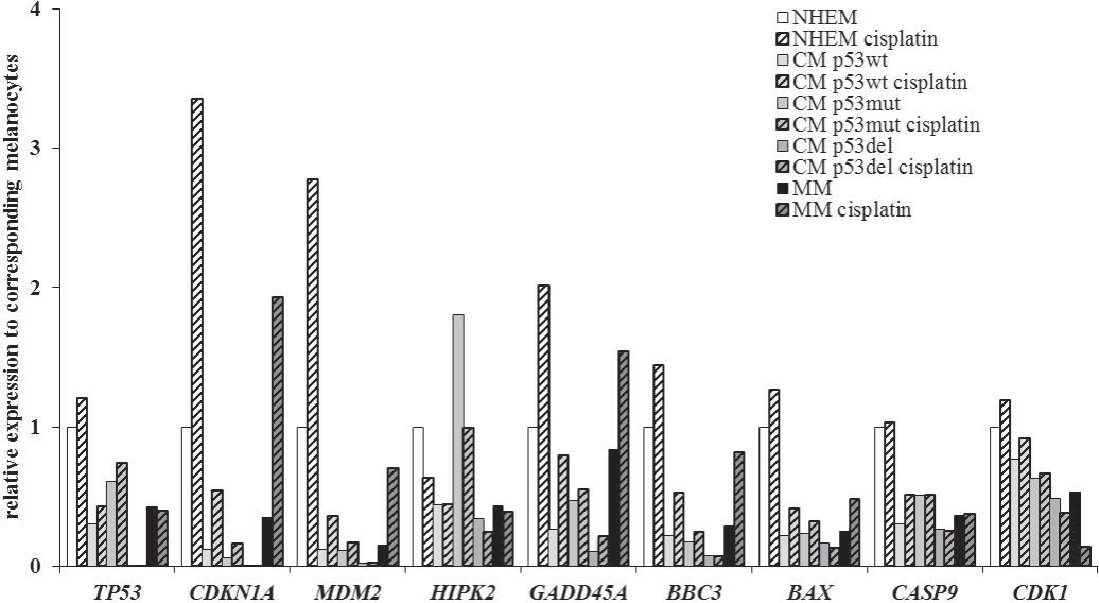
Quantitative PCR of the p53 pathway in primary cells and cell lines without treatment and after 24 h incubation with 8 μM cisplatin Results were normalized to GAPDH and shown as fold induction compared to CM. MM: Mucosal melanoma; CM: Cutaneous melanoma. Cis: Cisplatin.

QPCR of untreated MM cells and CM cell lines revealed different expression patterns of analysed genes due to the underlying p53 mutation status. Primary MM cells and melanoma cell lines harbouring wild-type p53 demonstrated a significantly lower *TP53* mRNA expression when compared with primary melanocytes. The lowest *TP53* mRNA levels were determined for p53-knockout cell line IGR-37. Parallel with *p53* mRNA levels, significantly decreased mRNA levels were detected for p53 stabilizing *HIPK2* in all p53 wild-type cell lines and IGR-37. No differences were observed for melanoma cell lines expressing mutated p53. Direct p53 target gene *CDKN1A* demonstrated significantly lower expressions for all tumour cell lines when compared with primary melanocytes. A significant and stepwise decrease can be observed in MM cells, from melanoma cell lines harbouring wild-type p53 to melanoma cell lines with mutated p53. *GADD45A* mRNA was expressed equally in primary melanocytes and MM cells; a significantly higher expression than that of CM cell lines. *BBC3* and *BAX* had significantly lower expression in MM and CM cells. The lowest levels demonstrated p53- knockout IGR-37. Analysis of *CASP9* demonstrated a significantly decreased mRNA expression when compared with primary melanocytes. No differences were observed for *CDK1* expression levels in MM and CM, except IGR-37 (Figure 3). To investigate the functional integrity of the p53 downstream, cells were treated with 8 μM cisplatin. There was no regulation of *TP53* in the analysed cell lines. All cell lines harbouring wild-type p53 demonstrated an upregulation of *MDM2*, with the highest expression in melanocytes and MM. *HIPK2* was downregulated in melanocytes and CM cell lines that expressed mutated p53. Cell cycle regulation *CDKN1A* was upregulated in all cell lines after treatment with cisplatin, while *GADD45A* was upregulated only in melanocytes. *BBC3* mRNA expression increased in CM cell lines with wild-type p53 and in MM. QPCR failed to demonstrate a regulation *BAX* or *CASP9*. A *CDK1* downregulation was exclusively seen in MM (Figure 3).

### Western blot of p53 and p53 target genes in cutaneous and mucosal melanoma cell lines

**Figure 4:**
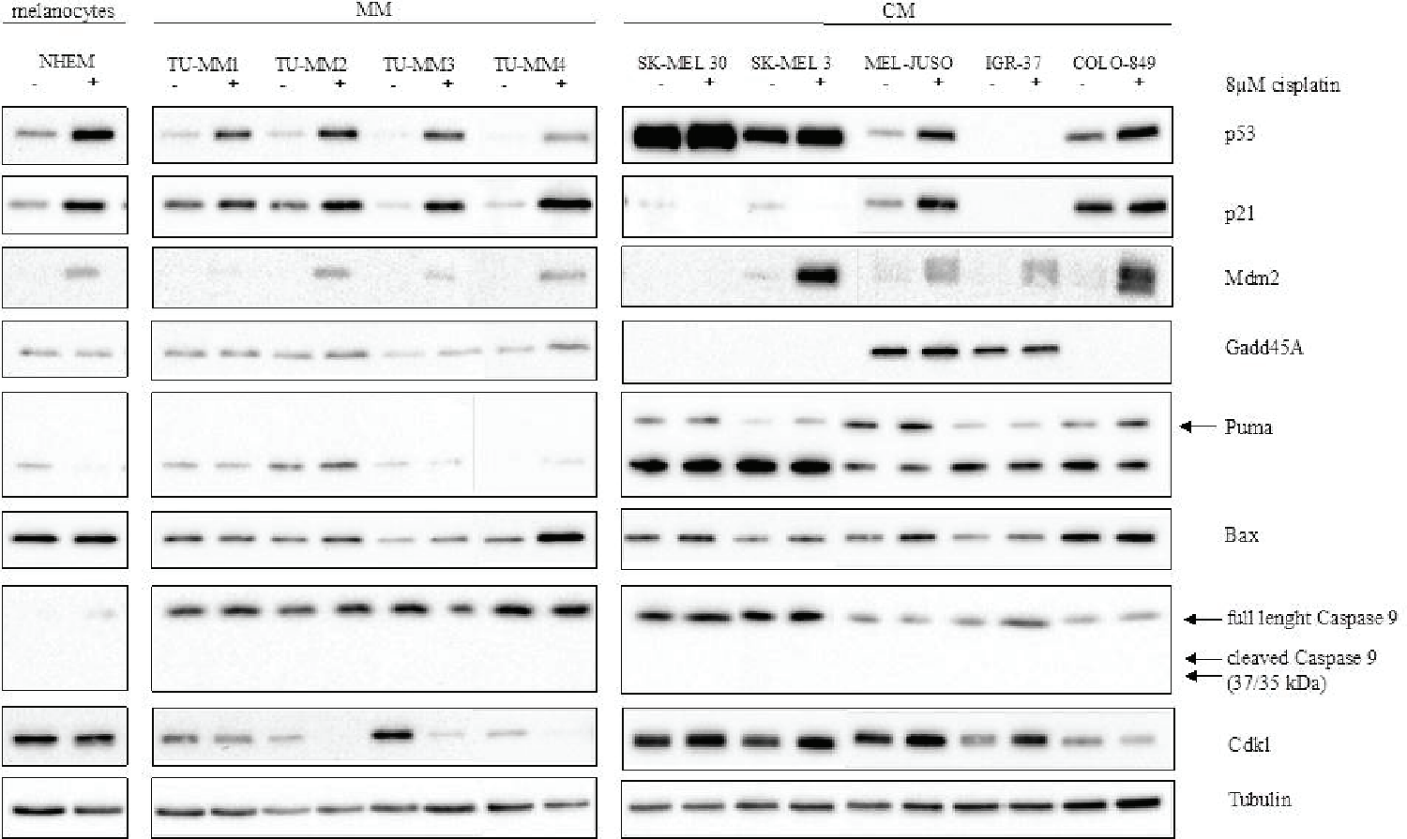
Cisplatin-induction of the p53 pathway in MM and CM cell lines Protein was isolated from cells treated with either fresh medium or 8 μM cisplatin for 24 hours. Tubulin served as loading control. MM: Mucosal melanoma; CM: Cutaneous melanoma. Cis: Cisplatin.

To further analyse the p53 activation capability after cisplatin treatment on protein level, the protein expression was analysed in western blot experiments. Despite unaltered *TP53* mRNA levels, a stabilisation of p53 protein could be observed in melanocytes and all MM cells. In SK-MEL 30, SK-MEL 3 and IGR-37 cisplatin incubation failed to induce the p53 protein. Cell lines that showed p53 induction demonstrated subsequent p21 upregulation. Independent of p53 or p21 expression, all cell lines, except SK-MEL30, showed an increased Mdm2 level, even though the protein amount of MM cells was difficult to detect. Treatment with cisplatin failed to induce a consistent response in the p53 downstream signalling. If detectable, Gadd45A and Puma showed no significant regulations. Bax stabilisation was seen in three out of four MM cells and weakly, but not significant, in all CM cells excluding COLO-849. MM and CM cells tended to regulate the cell-cycle regulating protein Cdk1 in a different manner. While Cdk1 protein expression is downregulated by cisplatin in MM cells, it is enhanced in CM cells. All untreated cells demonstrated Casp9 expression. Cells did not regulate Casp9 after treatment with cisplatin, nor did they show any cleave products (Figure 4).

## DISCUSSION

MM represents a small subgroup of malignant melanoma. The vast majority of MM originate in the sinonasal region and are clinically aggressive. This study includes 112 patients with CM and 27 with MM of the head and neck region. The high percentage of MM can be attributed to its sinonasal and oral origin and the subsequent admission to our otorhinolaryngology department. Similar to findings in the current literature, our study showed that patients with MM were significantly older than their cutaneous counterparts (MM 70 years, SD: 12, CM 58 years, SD: 15, *p* < 0.001) [4]. The prevalence of women was not determined in this study. According to the UICC staging system, most MM were classified as T3 tumours. At time of diagnosis no difference was seen in the locoregional metastasis rate (MM 7%, CM 13%, *p* < 0.396), but MM did demonstrated a tendency for less frequent distant metastases at the time of diagnosis (*p* < 0.064). A significant difference was seen in the disease-free and overall survival in favour of CM (*p* < 0.001). A multifocal tumor expansion and the difficult anatomic site in the sinonasal system often prevent resections with an appropriate R-status. Therefore, recurrence rates are estimated to be about 50%–90% [22]. While systemic therapeutic approaches failed to improve patients' prognoses in MM, it remains unclear whether the aggressive phenotype refers to a limited resectability in the sinonasal region or to distinct biological mechanisms. *TP53* is frequently mutated in solid tumours, whereas mutations in CM and MM are rare [12]. The mutation load in MM is estimated to be five- to ten-fold smaller than in CM [23]. Although protein stabilising mutations are rare, an accumulation of wild-type p53 can be demonstrated in the majority of CM and MM [24]. Recently, a disruption of cell-cycle regulating proteins, Bcl-2, p53 and p16, were associated with the carcinogenesis of primary oral mucosal melanomas [25]. While wild-type p53 demonstrates a short half-life of approximately 30 minutes, p53 is physiologically stabilised after cellular stresses by a broad variety of posttranslational modifications, including acetylation, phosphorylation and sumoylation, increasing its transcriptional activation, or enhancement of DNA and promoter specific binding [26, 27]. An accumulation of the p53 protein in untreated cells implies a disruption of its functional integrity indicating a p53 mutation or an interaction with viral oncogenes [28]. In the current cohort, all MM cells showed wild-type p53, while two mutations were detected in five CM cell lines. In contrast, western blot analysis demonstrated an accumulation of the p53 protein in all cells, except p53-knockout IGR-37. Immunohistochemical staining of 40 tumour samples confirmed the high p53 expression level in CM and MM. In our cohort, CM (92%) showed a significantly higher p53 expression level than MM (72%; *p* < 0.04). In the absence of protein stabilising mutations in seven of nine tumour cell lines, other mechanisms have to be considered to explain the abrogation of the p53 pathway, particularly the induction of apoptosis, resulting in the accumulation of wild-type p53. Blagosklonny constitutes a regulatory p53 feedback loop in which the loss of p53 function results in a ‘compensatory’ p53 upregulation by decreasing its degradation [28]. Therefore, the key point that leads to the disruption of the p53 pathway has to be assessed in our cohort. Recently, we demonstrated a functional p53 upstream in different melanoma cell lines [21]. In the current cohort, western blot experiments revealed cisplatin-induced p53 upregulation in all p53 wild-type cells, except SK-MEL 30. The high p53 base level did not result in further induction. Mdm2 and Hipk2 play a pivotal role in the p53 stabilisation via phosphorylation at serine residues. Overexpression of *MDM2* by enhanced protein translation was frequently observed in solid tumours and was associated with tumour progression [29–31]. However, qPCR experiments demonstrated a significantly higher expression of *Mdm2* mRNA expression of primary melanocytes when compared with CM and MM. QPCR analysis of FFPE samples and cells revealed a significantly higher *MDM2* mRNA expression in MM when compared with CM. Different qPCR levels did not result in a different protein level in IHC or WB analysis, most likely due to posttranslational mechanisms or a *TP53*-independent Mdm2 regulation. For example, a positive feedback loop is described for p53-induced Caspase2-mediated Mdm2 cleavage [32]. In addition, regardless of the *TP53* mutation status, *Mdm2* mRNA expression was upregulated after cisplatin treatment for CM and MM. Importantly, the Mdm2 up-regulation of IGR-37 suggested *TP53* independent mechanisms. The hepatocyte growth factor receptor and the insulin like growth factor 1 receptor were found to regulate *MDM2* translation by signalling through PI3K and mTOR. Furthermore, several RNA binding proteins were shown to interfere with *TP53* and *MDM2* [29]. In contrast, CM with mutated p53 showed a significantly higher *HIPK2* mRNA expression compared with wild-type cells. Pointing to an enhanced mutational load in CM, IHC staining of 40 tumour samples validated the higher expression in favour for CM [21, 23]. Cell lines of a different mutational status failed to demonstrate a significant *HIPK2* mRNA regulation after cisplatin treatment, which points to regulatory mechanisms on protein levels. In agreement with the current literature, we hypothesized a loss of p53 function in SK-MEL 30 which resulted in an incapability to induce Mdm2, p21 and Gadd45a [27]. This most likely indicates an interaction with viral oncoproteins or cellular proteins that stabilise non-functional wild-type p53 [28]. *GADD45A,* which is involved in DNA repair and cell cycle regulation, demonstrated inconclusive expression patterns. In FFPE samples, qPCR and IHC detected a significantly higher expression in CM, while cell experiments showed opposite results. Untreated CM cell lines expressed Gadd45a in two of five cases, including p53-knockout IGR-37, suggesting *TP53*-independent mechanisms. FoxO3a, Egr-1, c-myc and ZBRK have been identified as modulators of Gadd45a expression [29]. *CDK1* represents another cell cycle regulation gene that is involved in the G2/M arrest. Immunohistochemical staining, WB analysis and qPCR of FFPE samples and cells revealed no differences in the constitutional *CDK1*/Cdk1 expression. Interestingly, after treatment with cisplatin, only MM cells decreased mRNA and protein expression of *CDK1*/Cdk1, whereas CM cells tended to stabilize Cdk1 protein and therefore promote the maintenance of cell proliferation. Recently, the influence of epigenetics in cutaneous melanoma gained in importance. It was demonstrated that gene-specific hypermethylation silences genes involved in cell cycle regulation, DNA repair, apoptosis and cell signalling [33]. The potential impact of a distinct *CDK1* mediated cell cycle regulation in MM and CM remains elusive at this point. Immunohistochemistry of FFPE samples and tumour cells showed that the constitutional p53 expression resulted in a significantly higher *CDKN1A* expression in MM when compared with CM. Different *CDKN1A* mRNA levels did not result in a different constitutional protein expression as observed in IHC and WB. With respect to Blagosklonny's constitute of a compensatory upregulation of wild-type p53 due to functional loss, elevated *CDKN1A* mRNA levels are congruent with an accumulation of wild-type p53 but do not inevitably refer to a more ‘physiological’ behaviour, because a disruption of the apoptotic cascade will result in an increased regulatory feedback. *TP53* downstream genes *BBC3*, *BAX* and *CASP9* showed no difference in the mRNA expression in FFPE samples and untreated cells, except for a higher *BAX* expression for MM in FFPE samples. These results were verified on protein level. Immunohistochemical analysis of staining patterns showed no significant differences between the groups. Interestingly, despite a detectable mRNA level in FFPE and cell lines, MM failed to detect Puma in cell lines by WB experiments. Puma expression correlates inversely with the melanoma malignancy grade, and weak Puma expression is associated with poorer overall survival, suggesting Puma as a marker for disease aggressiveness [35, 36]. Recent studies demonstrated the regulation of Puma by oncogenic miRNAs [34]. In human oral squamous carcinoma cells, Puma was a direct target of miR-222 and mediated the diminished apoptosis after treatment with cisplatin [35]. Additionally, Puma degradation in melanoma cells could be inhibited with chloroquine, promoting apoptosis and demonstrating a significant role of Puma in apoptosis of melanoma cells [36]. In support of these results, we were not able to detect an upregulation of *CASP9* mRNA or protein after cisplatin treatment. Recently, the impact of Puma was highlighted in *BRAF (V600E)* melanoma cell lines. Unexpectedly, treatment with *MEK* inhibitors led to an inhibition of cisplatin-induced apoptosis in some cell lines via activation of the *PI3K/AKT* pathway [37]. The *PI3K-AKT* pathway was demonstrated to contribute to melanoma resistance as well as its tumour initiation [38]. While mutations in *BRAF* occur infrequently in MM, pAkt and pErk expression were demonstrated in sinonasal MM [9, 39]. Interestingly, primary melanocytes do not regulate pro-apoptotic proteins after cisplatin treatment. This phenomenon is described for many normal cell types, which are intrinsically apoptosis-resistant and results in chemo-resistant tumours after malignant transformation [40]. This may explain why melanoma is resistant to the majority of chemotherapeutics [41]. Furthermore, Apaf-1 inactivation is described for melanoma cells, supporting this hypothesis [42]. However, the disruption of Puma mediated apoptosis in MM may contribute, despite the challenging anatomic location, to the aggressive phenotype. Therapeutic strategies inhibiting the *PI3K-Akt-mTOR* pathway or activating the apoptotic capability of Puma are of major clinical import [39, 43].

## CONCLUSIONS

Our study confirmed the highly aggressive phenotype of MM. Comprehensive analysis of the p53 pathway in MM and CM revealed no differences in *TP53* and direct targets *CDKN1A* and *GADD45A* in wild-type cells that represent the majority of MM and CM. The regular induction of direct *TP53* target genes failed to induce apoptosis mediated by *CASP9*. A disruption of *BBC3* mediated apoptosis may result in the poor response of MM after treatment with cisplatin, and developing therapeutic approaches is of major clinical import. The potential influence of a distinctive *CDK1-*dependent cell cycle regulation has to be further investigated.

## MATERIALS AND METHODS

### Patient selection

The study included 139 patients with malignant melanoma of the head and neck, including 112 patients with CM and 27 patients with MM. Tumor samples were obtained from the tissue collection of the Institute of Pathology at the Technical University Munich. The tissue collection was approved by the local ethical committee. Tumor samples were histologically reviewed by at least two experienced pathologists. Clinical parameters and survival data were retrospectively collected including age at diagnosis, sex, TNM-staging, recurrence, death and loss to follow-up. Patients with lacking data, incomplete staging, and refused or unfinished treatment were excluded from survival analysis. The mean follow-up time was 89 months (range: 0–408 months). Paraffin-embedded tumour (FFPE) samples from 16 CM and 24 MM were randomly selected and analysed with qPCR and immunohistochemistry (IHC).

### Statistical analysis

Differences between both groups were analysed using the Chi square test and Fisher exact test for categorical and the unpaired student's *t*-test for continuous variables. As main endpoints the overall survival (OS) and recurrence-free interval (RFI) were assessed measuring the time from treatment to locoregional recurrence and death, and/or distant metastasis. Survival rates by the log-rank test for univariate analysis with *p*-values < 0.05 were considered statistically significant (SPSS Inc., Chicago, IL).

### Mammalian cell culture and treatment

Primary cells were obtained from mucosal melanoma tumour tissue. The tumour tissue was cut into little pieces and dried on culture dishes upside down for 30 minutes before medium was added. After three to four days, the tissue was removed and adhered mucosal melanoma cells were cultured. The mucosal melanoma primary cells TU-MM1–TU-MM4, as well as the melanoma cell lines SK-MEL 30, IGR-37, MEL-JUSO, COLO-849 and SK-MEL 3 (DSMZ, Braunschweig, Germany) were cultured under standard conditions (37°C, 5% CO_2_, fully humidified atmosphere) in Dulbecco's Modified Eagle's Medium or Roswell Park Memorial Institute 1640 medium (TU-MM3, TU-MM4, SK-MEL3), supplemented with 10% foetal calf serum (PAA Laboratories, Cölbe, Germany), 1% penicillin-streptomycin and 1% glutamine (all from Biochrom, Berlin, Germany). Normal human epidermal melanocytes (NHEM) were cultured in Melanocyte Medium 2 (PromoCell, Heidelberg, Germany). Cells were treated with 8 μM cisplatin (Teva, Ulm, Germany) for 24 hours before protein or RNA was isolated.

### RNA extraction from cells and FFPE

Cells were harvested from culture dishes and RNA was prepared using the RNeasy-Kit (Qiagen, Hilden, Germany) according to the manufacturer's instructions. RNA from FFPE was isolated using the High Pure FFPE RNA Micro Kit (Roche, Mannheim, Germany). The RNA concentration and purity was determined with the NanoDrop system (Thermo Scientific, Wilmington, USA).

### DNA extraction from cells and p53 sequencing

Cells were harvested from culture dishes and DNA was isolated using the DNeasy-Kit (Qiagen) according to the manufacturer`s instructions. DNA concentration and purity was determined with the NanoDrop system (Thermo Fischer). For sequencing (Eurofins, Ebersberg, Germany) *TP53* exons 5–8 were amplified with KAPA SYBR^®^ FAST (Kapa Biosystems, Woburn, United States) using 60 ng DNA. The following *TP53* primers were used: exon5 for 5′-atctgttcacttgtgccctg, rev 5′-aaccagccctgt cgtctctc, exon 6 for 5′-agggtccccaggcctctgat, rev 5′-cacccttaacccctcctccc, exon7 for 5′-ccaaggcgcactg gcctcatc, rev 5′-cagaggctggggcacagcagg, exon8 for 5′-ttccttactgcctcttgctt, rev 5′-tgtcctgcttgcttacctcg.

### Quantitative real-time PCR

250 ng of total RNA was reverse transcribed using the M-MLV reverse transcriptase (Invitrogen, NY, USA) or the QuantiTect Reverse Transcription Kit (Qiagen) for cultured cells or FFPE sections, respectively. QPCR was performed using KAPA SYBR^®^ FAST (Kapa Biosystems) and primers from the QuantiTect Primer Assay (Qiagen). Results were evaluated using the 2^−ΔΔCT^ method. A fold difference equal or larger than 2 was estimated to be statistically significant.

### Protein extraction and western blotting experiments

Total protein extraction was performed using cell lysis buffer (Cell Signaling Technology, Frankfurt, Germany) according to the manufacturer's instructions. The protein concentration in the supernatant was determined with a Bradford assay. Western blotting was performed using 15 μg protein per sample, which were separated by SDS-polyacrylamide gel electrophoresis and transferred on a polyvinylidene fluoride membrane (Carl Roth, Karlsruhe, Germany) by electroblotting. Antibodies were incubated in 5% non-fat milk in TBST (0.1% Tween-20, 20 mM Tris, 140 mM NaCl, pH 7.6) overnight at 4°C. Proteins were detected with antibodies against p53 (DO-7, Dako Deutschland GmbH, Hamburg, Germany), p21 Waf1/Cip1 (12D1), Puma (D30C10), Gadd45a (D17E8), Bax (D2E11), CDK1 (8G10) (Cell Signaling Technology, Danvers MA, United States) and Mdm2 (SMP14, Santa Cruz, Heidelberg, Germany) using the biotechnology SuperSignal West Pico Chemiluminescent Substrate (Thermo Fisher, Rockford IL, USA).

### Immunohistochemistry

FFPE tumour sections (2.5–3 μm) were stained with antibodies against p53 (DO-7, Dako), p21 (9L524), GADD45A, HIPK2, MDM2 (US Biological, Massachusetts, USA), CDK1 (EPR165), PUMA (EP512Y), BAX (E63) (Epitomics, Burlingame, CA, USA) and visualized with the Bond Polymer Refine Detection Kit (Leica, Nussloch, Germany). A positive staining was defined as greater than 10% stained cells. Tissues with known expression of the respective antigens were used as positive controls.
